# Disynaptic specificity of serial information flow for conditioned fear

**DOI:** 10.1126/sciadv.abq1637

**Published:** 2023-01-18

**Authors:** Léma Massi, Kenta M. Hagihara, Julien Courtin, Julian Hinz, Christian Müller, Maria Sol Fustiñana, Chun Xu, Nikolaos Karalis, Andreas Lüthi

**Affiliations:** ^1^ Friedrich Miescher Institute for Biomedical Research, Maulbeerstrasse 66, Basel CH-4058, Switzerland.; ^2^ University of Basel, Basel, Switzerland.; ^3^ Institute of Neuroscience, Center for Excellence in Brain Science and Intelligence Technology, Chinese Academy of Sciences, Shanghai, China.

## Abstract

Memory encoding and retrieval rely on specific interactions across multiple brain areas. Although connections between individual brain areas have been extensively studied, the anatomical and functional specificity of neuronal circuit organization underlying information transfer across multiple brain areas remains unclear. Here, we combine transsynaptic viral tracing, optogenetic manipulations, and calcium dynamics recordings to dissect the multisynaptic functional connectivity of the amygdala. We identify a distinct basolateral amygdala (BLA) subpopulation that connects disynaptically to the periaqueductal gray (PAG) via the central amygdala (CeA). This disynaptic pathway serves as a core circuit element necessary for the learning and expression of conditioned fear and exhibits learning-related plasticity. Together, our findings demonstrate the utility of multisynaptic approaches for functional circuit analysis and indicate that disynaptic specificity may be a general feature of neuronal circuit organization.

## Teaser

A specific di-synaptic amygdala-to-brainstem pathway is necessary for the learning and expression of conditioned fear.

## INTRODUCTION

Learning to associate potential threats with predictive cues is an adaptive brain function critical for survival. Previous work has identified basolateral amygdala (BLA) and central amygdala (CeA) (*1*) as two indispensable brain structures for the acquisition and expression of conditioned fear memories. On the basis of anatomical tracing studies (*2*) and pharmacological (*3*) and optogenetic manipulations (*4*, *5*), a canonical model has been developed in which conditioned sensory inputs are transmitted serially from the BLA to the CeA and eventually to the ventrolateral periaqueductal gray (vlPAG), a midbrain structure involved in the generation of conditioned defensive behaviors, such as freezing (*6*–*10*). However, there remains scant, controversial experimental evidence for the role of this pathway from the BLA to the vlPAG, as opposed to alternative (*11*, *12*) or more complex indirect pathways (*13*) in the acquisition and expression of conditioned appetitive and aversive behaviors (*5*, *13*, *14*). Here, we combined transsynaptic tracing, optogenetic manipulations, and fiber photometry to directly examine the functional organization and behavioral relevance of this disynaptic pathway connecting the BLA to the vlPAG via the CeA.

## RESULTS

We first sought to describe the anatomical organization of the BLA-CeA-vlPAG pathway. To achieve this, we used a disynaptic rabies virus (RV) tracing approach. We injected a retrograde virus encoding cre into the vlPAG and a red fluorescent protein–encoding EnvA pseudotyped G protein–deleted RV into the CeA (fig. S1A). We found disynaptically rabies-labeled neurons predominantly located in the posterolateral part of the BLA (BLAp; fig. S1, B and C). Although a recent study has systematically investigated BLAp outputs and found various subcortical projections, including the lateral septum, bed nucleus of the stria terminalis, and mediodorsal thalamic nucleus (*15*), its projections to the CeA have not been addressed. Earlier tracing studies described that both the BLAp and anterior BLA (in particular, basal amygdala) send axons to the medial CeA (*2*, *16*, *17*), suggesting that conventional axon tracing methods are not sufficient to describe the anatomical specificity of the BLAp. Notably, as previously described, we found no direct projection from the BLA to the vlPAG (fig. S1, D to F) (*18*).

**Fig. 1.**
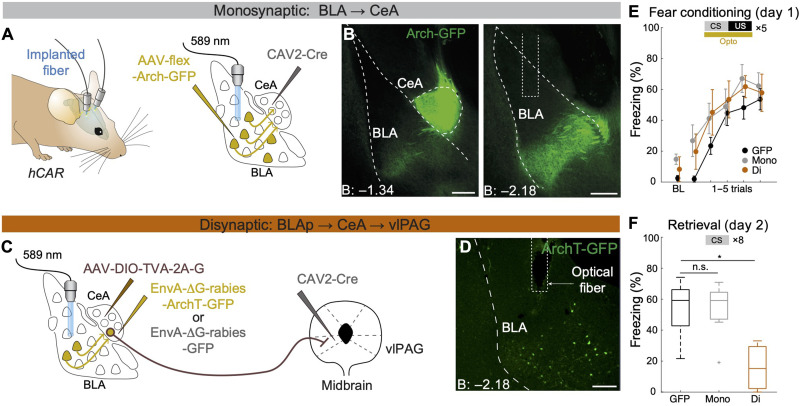
Disynaptic specificity of BLAp→CeA→vlPAG pathway for conditioned fear. (**A**) Scheme illustrating viral injection strategy to express Arch in neurons projecting from the BLA to CeA. *CAV2-Cre* was injected into the CeA, and then, AAV-flex-Arch-GFP was injected into the BLA. Optic fibers were bilaterally implanted to target labeled BLA neurons. hCAR mice were used to facilitate transduction by CAV2 viruses. (**B**) Histology of injection and fiber implantation sites: (left) CeA and (right) BLA. Scale bars, 250 μm. (**C**) Scheme illustrating viral injection strategy to express ArchT in neurons projecting from the BLAp to the CeA-vlPAG pathway. *CAV2-Cre* was injected into the vlPAG and AAV-DIO-TVA-2A-G was injected into the CeA in hCAR mice. Three days before FC, rabies-ArchT-GFP or rabies-GFP was injected into the CeA. Optic fibers were bilaterally implanted to target posterolateral BLA neurons. (**D**) Histology of the fiber implantation site. Scale bar, 200 μm. (**E**) Average freezing levels (means ± SEM) during auditory FC (day 1). Optogenetic manipulation was applied to cover CS + US periods. Freezing levels during a 2-min baseline (BL) and during each CS presentation (trials 1 to 5). *N* = 13, 10, and 7 mice for GFP (GFP control group), Mono (monosynaptic manipulation group), and Di (disynaptic manipulation group), respectively. (**F**) Average freezing levels (means ± SEM) during eight CS presentations during fear memory retrieval (day 2). *P = 0.0001; not significant (n.s.) *P* = 0.99; Dunnett’s test.

Before investigating the functional role of this disynaptic BLAp-CeA-vlPAG pathway, we wanted to assess the involvement of the monosynaptic projection from BLA neurons to anatomically unspecific CeA neurons in the acquisition and expression of conditioned freezing responses. To achieve this, we optogenetically inhibited BLA neurons projecting to the CeA using an intersectional viral approach (Fig. 1; see Materials and Methods). Inhibition of CeA-projecting BLA neurons during auditory fear conditioning (FC) had no effect on freezing behavior during FC (Fig. 1E) or, consistent with previous work (*5*), during fear memory retrieval on the next day (Fig. 1F and fig. S6B). Because BLA neurons are functionally highly heterogeneous, we hypothesized that the simultaneous inhibition of anatomically undefined CeA-projecting BLA neurons might have masked the function of specific pathways involved in the acquisition and expression of conditioned fear. Therefore, we next tested whether manipulating the subset of BLA neurons that form the BLAp-CeA-vlPAG disynaptic pathway would have a distinct effect on fear learning. To achieve this, we used disynaptic RV tracing with ArchT-encoding RV (Fig. 1, C and D; see Materials and Methods). When we optogenetically inhibited the BLAp-CeA-vlPAG pathway during FC, we found no effects on acute freezing behavior during FC (Fig. 1E) but found a marked deficit in memory retrieval on the next day (Fig. 1F and fig. S6B). These effects could not be attributed to RV-associated toxicity because, in the absence of photosilencing, rabies-infected animals could be reconditioned (fig. S2).

**Fig. 2.**
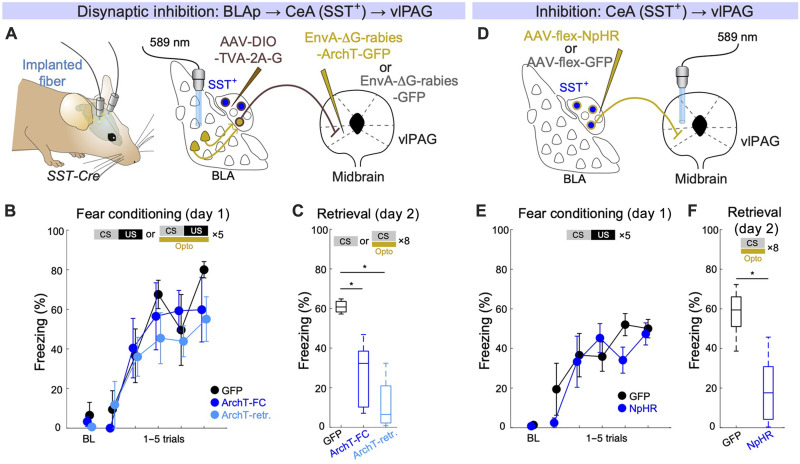
SST^+^ CeA neurons connect BLAp to vlPAG. (**A**) Scheme illustrating viral injection strategy to express ArchT in neurons projecting from the BLAp to the CeA(SST^+^)-vlPAG pathway. AAV-DIO-TVA-2A-G was injected into the CeA in *SST-Cre* mice, and then, rabies-ArchT-GFP or rabies-GFP was injected into the vlPAG. Optic fibers were bilaterally implanted to target BLAp. Experimental animals underwent optogenetic manipulations during either FC (ArchT-FC) or retrieval (Arch-retrieval). GFP controls had the laser on both during FC and retrieval. Optogenetic manipulations were performed to temporally cover CS + US or CS presentations. (**B**) Average freezing levels (means ± SEM) during auditory FC (day 1). Freezing levels during a 2-min baseline and during each CS presentation (one to five trials). For the ArchT-FC group, optogenetic manipulation was applied to cover CS + US periods during FC but not during CS presentation during retrieval. In contrast, for the ArchT-retrieval group, optogenetic manipulation was applied to cover CS periods during retrieval but not during CS + US presentation during FC. *N* = 4, 5, and 4 mice for GFP, ArchT-FC, and ArchT-retrieval, respectively. (**C**) Average freezing levels (means ± SEM) during eight CS presentations in fear memory retrieval (day 2). ArchT-FC versus GFP: *P = 0.012; ArchT-retrieval versus GFP: *P = 0.002; Dunnett’s test. (**D**) Scheme illustrating viral injection strategy to express ArchT in CeA(*SST*^+^) neurons. AAV-flex-NpHR was injected into the CeA in *SST-Cre* mice. Optic fibers were bilaterally implanted to target vlPAG. (**E**) Average freezing levels (means ± SEM) during auditory FC (day 1) without optogenetic manipulation. *N* = 5 and 8 for GFP and SST-NpHR, respectively. (**F**) Average freezing levels (means ± SEM) during eight CS presentations in fear memory retrieval (day 2). Optogenetic manipulations were performed to temporally cover CS presentations. *P = 0.0031; rank sum test.

Although these experiments revealed the role of this disynaptic pathway in fear learning, the cell type specificity of the pathway remained unclear. The CeA contains distinct genetic marker–defined neuronal subpopulations that exhibit distinct functional roles and activity profiles (*10*, *13*, *19*). Previous work identified somatostatin-expressing CeA neurons (SST^+^) as a major subpopulation projecting to the vlPAG (*8*, *13*), and stimulation of SST^+^ CeA neurons elicits freezing behavior in naïve and conditioned animals (*19*, *20*) [but see (*13*)]. However, whether this effect is mediated by direct projections of SST^+^ CeA neurons to the vlPAG has not been tested to date. Using cell type–specific RV tracing, we established that SST^+^ CeA neurons projecting to the vlPAG receive inputs from BLAp neurons (Fig. 2A and fig. S3; see the Supplementary Materials). We rarely found rabies-positive neurons in the medial part of the BLA, which is juxtaposed to the CeA, suggesting that Cre-dependent adeno-associated virus (AAV) injections targeting SST^+^ CeA neurons did not cause leakage into SST^+^ BLA neurons (fig. S3E). Using ArchT-mediated optogenetic inhibition during FC, we showed that this disynaptic pathway is indispensable both for the acquisition and for the retrieval of conditioned freezing responses (Fig. 2, B and C, and fig. S6C). These effects are likely mediated by direct BLAp inputs onto SST^+^ CeA neurons projecting to the vlPAG, rather than due to axonal collaterals of SST^+^ CeA cells to other regions because optogenetic inhibition of the axonal terminals of SST^+^ CeA neurons in vlPAG recapitulated the behavioral effects of the disynaptic circuit inhibition (Fig. 2, D to F, and fig. S6D). Because of technical limitations, we cannot completely exclude the possibility that some BLAp neurons were labeled trisynaptically via intra-CeA SST^+^-SST^+^ connections (*21*). However, considering that activation of CeA to vlPAG projections is necessary for fear learning, trisynaptic labeling would be difficult to reconcile with the results of our optogenetic manipulations.

**Fig. 3.**
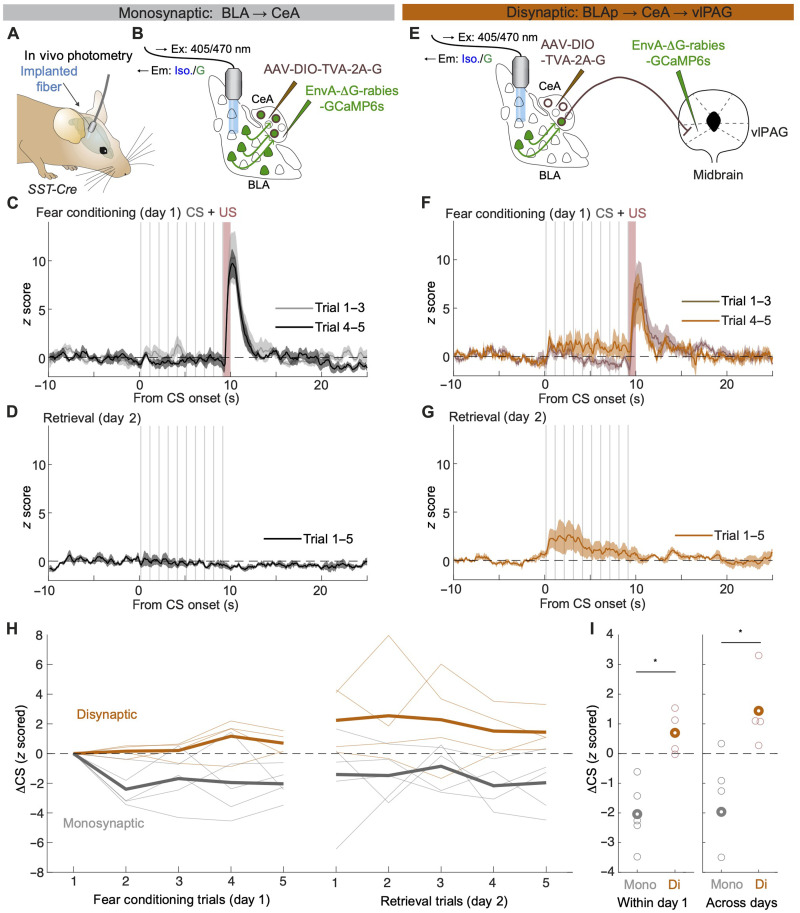
BLAp neurons that disynaptically connect to vlPAG show learning-related CS response plasticity. (**A** and **B**) Scheme illustrating viral injection strategy to express GCaMP6s in neurons projecting from the BLA to CeA. AAV-DIO-TVA-2A-G was injected into the CeA in *SST-Cre* mice, and then, rabies-GCaMP6s was injected into the CeA. Optic fibers were unilaterally implanted to target labeled posterior BLA neurons. (**C** and **D**) Averaged *z*-scored photometry traces on day 1 (C) or day 2 (D) from monosynaptically labeled mice. Means ± SEM (*N* = 5 mice). (**E**) Scheme illustrating viral injection strategy to express GCaMP6s in neurons projecting from the BLAp to the CeA(SST^+^)-vlPAG pathway. AAV-DIO-TVA-2A-G was injected into the CeA in *SST-Cre* mice, and then, rabies-GCaMP6s was injected into the vlPAG instead of CeA. Optic fibers were bilaterally implanted to target GCaMP6s-expressing BLAp neurons. (**F** and **G**) Averaged *z*-scored photometry traces on day 1 (F) or day 2 (G) from disynaptically labeled mice. Means ± SEM (*N* = 4 mice). (**H**) Summary of responses to CSs. CS responses to the first CS on day 1 were subtracted from those to each CS. Thick lines show group averages. Thin lines represent data from individual animals. (**I**) CS response comparisons within day 1 (left) and across days (right). Thick circles show group averages. Thin circles represent data from individual animals. *P = 0.016 (left); *P = 0.032 (right); rank sum test.

Last, to selectively monitor the activity dynamics of BLAp neurons projecting onto SST^+^ CeA neurons during FC and memory retrieval, we used GCaMP6s-encoding RV and fiber photometry (Fig. 3 and fig. S4). We found that, on the population level, BLAp neurons projecting to undefined SST^+^ CeA neurons showed robust unconditioned stimulus (US) responses but did not show conditioned stimulus (CS) responses during conditioning or retrieval (Fig. 3, A to D). In stark contrast, BLAp neurons projecting to vlPAG-projecting SST^+^ CeA neurons not only responded to the US but also developed CS responses during FC that remained stable during memory retrieval on the next day (Fig. 3, E to I, and fig. S6, E and F). Single-neuron resolution Ca^2+^ imaging using a miniaturized microscope revealed that target-nondefined BLAp neurons showed bidirectional CS responses, similar to observations made in anterodorsal BLA (fig. S5) (*22*). Thus, the lack of population CS responses in the BLAp neurons projecting to the target-nondefined population of SST^+^ CeA neurons reflects CS response heterogeneity of individual BLAp neurons, while the disynaptic target-defined BLAp subpopulation exhibits a more homogeneous learning-induced CS response increase.

## DISCUSSION

By combining the anatomical specificity of transsynaptic tracing with functional recordings and optogenetic manipulations, we demonstrate that the acquisition and expression of conditioned fear memories are mediated by a core circuit consisting of a disynaptic projection linking an anatomically defined subpopulation of neurons in the posterior BLA to the vlPAG via SST^+^ neurons in the CeA. This indicates an exquisite level of anatomical and functional specificity of BLA neurons and identifies the BLAp as a key structure in FC. Our single-cell resolution miniscope imaging revealed that BLAp neurons are a functionally heterogeneous population, containing not only neurons that gain CS responses during learning but also others that lose their CS responses (fig. S5). On average, this functional heterogeneity likely resulted in the absence of any obvious CS response when we recorded population calcium dynamics using fiber photometry (Fig. 3). Because regulating the balance of activity between functionally opposing populations can be a powerful circuit mechanism for dynamically defining and switching an animal’s fear state (*19*, *23*), this could also explain the lack of effect of the monosynaptic optogenetic manipulations (Fig. 1). In the current study, we were not able to combine rabies-based GCaMP expression with single-neuron resolution imaging because of technical difficulties (see Materials and Methods). With improved RV tools (*24*–*26*), it would be possible to further characterize heterogeneity within mono- or disynaptically labeled BLA subpopulations.

Future investigations will have to address whether and to what extent BLAp is an important site of plasticity in the BLAp-CeA-vlPAG pathway. Furthermore, our data raise important questions regarding upstream brain areas processing CS- and US-related information that feed into the BLAp-CeA-vlPAG pathway and the nature of interactions between this and other neural circuits, such as the parabrachial-CeA pathway (*11*, *12*) and a neuronal population located in the medial CeA that exerts disinhibitory control over vlPAG (*4*, *27*). In addition, other cell types in the CeA such as PKCδ- or corticotropin-releasing factor–expressing neurons (*27*) or other transcriptionally defined neuronal subpopulations (*28*) could potentially serve as alternative nodes that connect the BLA to the vlPAG. How these locally intermingled and interacting information streams enable behavioral output awaits further investigation. On a more general level, our results indicate that functional analysis of a neuronal pathway should take into account not only the target area but also the genetic identity (*29*) as well as the anatomical projection targets of the postsynaptic neurons.

## MATERIALS AND METHODS

### Animals

*SST-ires-Cre* (*30*), *hCAR* (*31*), *tdTomato reporter* (*Ai14*), and wild-type C57/BL6J (Harlan/Envigo) mice were used. Genetically modified mice were backcrossed to a C57BL/6J background for at least seven generations. Mice were individually housed for at least 2 weeks before starting behavioral paradigms in open cages. Littermates of the same sex were randomly assigned to experimental groups. For behavioral experiments, only male mice (aged 2 to 3 months at the time of injection) were used. Male and female mice (aged 2 to 4 months at the time of injection) were used for rabies tracings. These analyses indicated no discernible differences between males and females. Room temperature was set at 22°C (±2°C), and room humidity was set at 55% (±10%). Mice were kept in a 12-hour light/12-hour dark cycle with access to food and water ad libitum. Behavioral experiments were performed during the light cycle, and all mouse procedures were performed in accordance with institutional guidelines at the Friedrich Miescher Institute for Biomedical Research and were approved by the Veterinary Department of the Canton of Basel-Stadt.

### Virus preparations

The *SAD*Δ*G* RVs were generated as described before (*18*, *32*) with a slight modification to achieve a higher titer (*33*). Briefly, Δ*G-mCherry* (*34*), Δ*G-GFP* (*35*), Δ*G-ArchT-GFP* (*18*), and Δ*G-GCaMP6s* (*33*) viruses were amplified from local viral stocks in B7GG cells [baby hamster kidney cells expressing T7 RNA polymerase, rabG, and histone-tagged green fluorescent protein (GFP)]. EnvA pseudotyped RVs were generated in baby hamster kidney–EnvA cells. The virus was concentrated by two rounds of centrifugation, suspended in Hanks’ balanced salt solution (Gibco), and titered in human embryonic kidney 293–TVA cells (provided by J. A. T. Young, Salk Institute) with serial dilutions of the virus. The titers of the EnvA pseudotyped rabies used for injections were in the range of 5 × 10^8^ to 1.0 × 10^10^ infectious units/ml. The viruses were stored at −80°C until further use. *CAV2-Cre* (*36*) virus was provided by E. Kremer (University of Montpellier, France). The titer was 3.1 × 10^12^ pp/ml. *AAV.EF1a.DIO.TVA950.2A.CVS11G* plasmid was a gift provided by K. Yonehara (*37*). It was packaged as AAV2/7 serotype at Vector BioLabs. *AAV.hSyn.flex.synaptophysin-EGFP* (*38*) was a gift provided by S. Arber. It was packaged as AAV2/1 serotype at Vector BioLabs.

### Stereotaxic surgeries

Buprenorphine (0.1 mg/kg of body weight; Temgesic, Indivior UK Limited) was injected subcutaneously 30 min before the surgery. Mice were anesthetized using isoflurane (5% for induction and 1 to 2% for maintenance; Attane, Provet) in oxygen-enriched air (Oxymat 3, Weinmann) and then head-fixed in a stereotaxic frame (model 1900, Kopf Instruments). Lidocaine and ropivacaine (lidocaine HCl, Bichsel; 10 mg/kg of body weight; Naropin, AstraZeneca; 3 mg/kg of body weight) were injected subcutaneously as local anesthesia before incision to the skin. Postoperative pain medication included buprenorphine (0.01 mg/ml in the drinking water, overnight) and injections of meloxicam (5 mg/kg, subcutaneously; Metacam, Boehringer Ingelheim) for 3 days after the surgery. Eyes were protected with ophthalmic ointment (Viscotears, Alcon). Rectal body temperature of the animal was monitored and maintained at 35° to 37°C using a feedback-controlled heating pad while fixed on the stereotactic frame.

### Viral injections and fiber/lens implantation

A volume of 50 to 200 nl of virus solution (depending on viral titer and area) was pressure-injected intracranially using calibrated glass pipettes (5-μl microcapillary tube, Sigma-Aldrich) connected to a Picospritzer III (Parker). For targeting the vlPAG, to avoid the subcranial midline blood sinus, craniotomies with a diameter of 0.3 mm were made bilaterally into the skull at ±1.7 mm from the midline suture and at the level of the lambda suture. The injection capillary was then slowly lowered using a hydraulic micropositioner (Kopf Instruments, model 2650) at a zenith angle of 26° to the target depth of 3 mm below the brain surface. Coordinates for CeA injections were as follows: anteroposterior (AP), −1.1 mm (from bregma); mediolateral (ML), ±2.7 mm (from bregma); and dorsoventral (DV), 4.2 mm (from pia). Coordinates for BLAp were as follows: AP, −2.18 mm (from bregma); ML, ±3.4 mm (from bregma); and DV, 4.2 mm (from pia).

For non–cell type–specific monosynaptic inhibition (Fig. 1A), *CAV2-Cre* and *AAV2/5.flex.Arch.GFP* (Penn Vector Core) were injected in the CeA and BLA, respectively. In the same surgery, optic fibers were implanted above the BLAp. Behavioral experiments were performed at the earliest 3 weeks after to ensure viral expression of the opsin. For non–cell type–specific disynaptic inhibition (Fig. 1C), *CAV2-Cre* and *AAV2/7.DIO.TVA950.2A.CVS11G* were injected in the vlPAG and CeA in the first surgery, respectively. Two to four weeks later, *EnvA-*∆*G-ArchT-GFP* or *EnvA-∆G-GFP* was injected in the CeA, and optic fibers were implanted above the BLAp. Two days after the second surgery, mice were subjected to behavioral experiments. For non–cell type–specific disynaptic tracing (fig. S1A), *CAV2-Cre* and *AAV2/7.DIO.TVA950.2A.CVS11G* were injected in the vlPAG and CeA in the first surgery, respectively. Two to four weeks later, EnvA-∆G-mCherry was injected into the vlPAG. For monosynaptic tracing from CeA to vlPAG (fig. S1D), *CAV2-Cre* was injected in the vlPAG of *hCAR::tdTomato* mice. For vlPAG injections, blue beads were coinjected with *CAV2-Cre* to confirm the injection site.

For cell type–specific disynaptic manipulations (Fig. 2A and fig. S4), *AAV2/7.DIO.TVA950.2A.CVS11G* was injected into the CeA of *SST-ires-Cre* mice. Two to four weeks later, *EnvA-∆G-ArchT-GFP* or *EnvA-∆G-GFP* was injected in the vlPAG to infect TVA-expressing axon terminals originating from SST^+^ neurons in the CeA. Optic fibers were then implanted above the BLAp. Some *EnvA-*∆*G-GFP* mice were fear-conditioned with light, subjected to memory retrieval without light and then to retrieval with light to serve as controls for both groups. For terminal inhibition of CeA (SST^+^) neurons (Fig. 2F), *AAV2/9.EF1a.DIO.NpHR3.3.EYFP* (Penn Vector Core) or *AAV2/5.flex.GFP* (UNC Vector Core) was injected into the CeA, and the optical fibers were placed into the vlPAG. *AAV2/1.flex.Synaptophysin.GFP* was used for anterograde tracing experiments (fig. S2A). For cell type–specific disynaptic tracing (fig. S2D), *AAV2/7.DIO.TVA950.2A.CVS11G* was injected into the CeA of *SST-ires-Cre* mice. Two to four weeks later, *EnvA-*∆*G-GFP* was injected into the vlPAG.

For photometry recordings (Fig. 3), *AAV2/7.DIO.TVA950.2A.CVS11G* was injected into the CeA of *SST-ires-Cre* mice. Two to four weeks later, *EnvA-*∆*G-GCaMP6s* was injected in the CeA (monosynaptic) or vlPAG (disynaptic), and an optic fiber was unilaterally implanted above the BLAp. Seven to nine days after the second surgery, recording experiments were executed.

Imaging with the miniature microscope (fig. S5) was attained by injecting *AAV.2/5.CaMKII.GCaMP6f* (*39*) (UPenn Vector Core) unilaterally into the BLAp. In the same surgery, a GRIN (graded-index) lens with 0.6 mm diameter (Inscopix) was implanted to the injection site. Three to four weeks after surgery, when the implanted GRIN lens got stabilized in the tissue, imaging experiments were started. Because RV-based GCaMP expression could start causing toxicity and cell death in synaptically infected neurons at around 1 week after infection, we did not use *EnvA-∆G-GCaMP6s* for miniature microscope imaging experiments.

### Behavioral paradigm

Two different contexts were used. Context A (retrieval context) consisted of a clear cylindrical chamber (diameter: 23 cm) with a smooth floor, placed in a dark-walled sound-attenuating chamber under dim-light conditions. The chamber was scented and cleaned with 1% acetic acid. Context B (FC context) contained a clear square chamber (26.1 cm by 26.1 cm) with an electrical grid floor (Coulbourn Instruments) for foot shock delivery, placed in a light-colored, sound-attenuating chamber under bright-light conditions and was scented and cleaned with 70% ethanol. A stimulus isolator (ISO-Flex, A.M.P.I.) was used for the delivery of DC shock. Both chambers contained overhead speakers for the delivery of auditory stimuli, which were generated using a System 3 RP2.1 real-time processor and SA1 stereo amplifier with RPvdsEx Software (all Tucker-Davis Technologies). Cameras (Stingray, Allied Vision) for tracking animal behavior were also equipped in both chambers. Radiant Software (Plexon) was used to generate precise TTL pulses to control behavioral protocols, and all the TTL signals, including miniscope frame timings, were recorded by Plex Control Software (Plexon) to synchronize behavioral protocols and behavioral tracking. On day 0, mice were habituated for 10 min to context A. On day 1 (FC session), mice were placed in context B and, after a 2-min baseline, subjected to five pairings of the CS (pure tones of 7.5 kHz, total duration of 10 s, consisting of 200-ms pips repeated at 0.9 Hz; 75-dB sound pressure level) presented five times with the US (2 s, DC of 0.65 mA applied back to back after the last pip). Animals remained in the context for 1 min after the last CS presentation and were then returned to their home cage. On day 2 (memory retrieval session), after a 2-min baseline period in context A, the CS was presented eight times (inter-trial interval of 35 to 60 s). For reconditioning experiments, on day 3, the experimental procedure was similar to the FC session, but a 3-kHz tone was used as CS (CS2); memory was evaluated 24 hours later with CS2 presentation. CS was generated using the timing of CS and US, where freezing behavior was quantified using Cineplex Editor (Plexon). The animals were considered freezing if no movement was detected for 2 s, and the measure was expressed as a percentage of time spent freezing.

### Optogenetic manipulations

All manipulations were performed with bilaterally implanted custom-made optic fiber connectors [FP200URT, 0.48 numerical aperture (NA), Ø200 μm; Thorlabs). Implants were fixed to the skull using cyanoacrylate glue (Ultra Gel, Henkel) and miniature screws (P.A. Precision Screws). Dental acrylic (Paladur, Heraeus) mixed with black paint was used to seal the skull. Implanted connectors were linked to a custom-built laser bench via optic fibers during behavior sessions with optogenetic manipulations. An acousto-optic tunable filter- (AA Opto-Electronic) was used to control the intensity and timing of lasers (MBL-589, 589-nm wavelength for Arch/ArchT and NpHR; CNI Lasers, China), which was triggered by TTL generated by Radiant Software (Plexon). The laser intensity was 11 to 15 mW at the end of the optic fiber.

### Fiber photometry recording

Recordings were performed with unilaterally implanted custom-made optic fiber connectors (FP400URT, 0.50 NA, Ø400 μm; Thorlabs). A modified Doric Fiber Photometry system (Doric) was used to perform the recordings as previously described (*23*). Briefly, two different excitation wavelengths were used (465 nm for Ca^2+^-dependent GCaMP6s activity and 405 nm to record an isosbestic, Ca^2+^-independent reference signal that serves to correct for photobleaching and movement-related artifacts). Data were preprocessed and analyzed using custom programs written in MATLAB (2017b, MathWorks). Data with obvious motion artifacts in the isosbestic channel were discarded. Demodulated raw Ca^2+^ traces were downsampled to 1 kHz and then detrended using a low-cut filter (Gaussian, cutoff of 2 to 4 min) to correct for slow drift of the baseline signal due to bleaching. Filtered traces were *z*-scored by the mean and SE of the entire trace. The behavioral paradigm was similar to what was described above, but the timing control of CS and US, video acquisition for animal behavior monitoring, and synchronization with photometry recording were achieved using custom-written Python and Bonsai (*40*) scripts (https://github.com/nikolaskaralis/OE_Bonsai_network_sync).

### Deep-brain Ca^2+^ imaging

For Ca^2+^ imaging using the miniature microscope (nVista HD, Inscopix), mice were prepared as previously described (*22*, *23*). Imaging data were acquired using nVista HD software (Inscopix) at a frame rate of 20 Hz. Time stamps of imaging frames and behavioral coordinates were collected for alignment using an Omniplex data acquisition system (Plexon), and imaging and video recording was triggered by Radiant (Plexon).

Data analysis was performed using custom codes written in MATLAB (2017b, MathWorks). Translational motion correction was performed by manual selection of two regions of interest (ROIs). To cope with the high background noise, imaging files were preprocessed by subtracting a Gaussian blurred image on a frame-by-frame basis. Motion correction was performed using fast Fourier transform–based image registration (*41*). A template was generated by registering the first 100 frames in the first ROI to the first frame and subsequently registering all images to the median image of the first 100 frames in the first ROI. This process was repeated until the applied motion fell under a specified value, typically less than 0.01 average pixel shift on all frames. Motion correction was then repeated on the second defined ROI to ensure that no (or only little) nonrigid motion was present in the recorded images. To avoid any interference with the subsequent extraction of calcium traces, the calculated shifts were applied to the raw data, and subsequent analyses were performed with the motion-corrected raw data.

Each session was processed independently, and CNMF-E (*42*) was used for automatic neuron extraction. Parameters were set to avoid false negatives at the cost of false positives, which were excluded through automated selection and visual inspection. ROIs were excluded if they were too small (approximately <0.6% of pixels), too big (approximately >1.4% of pixels), or too close to the edge of the field of view. In addition, neurons that exhibited low signal-to-noise ratios and overlapped at least 60% with other neurons were excluded from the analysis. Only ROIs that had coherent shapes, exhibiting round or slightly elongated contours, and showed clearly defined Ca^2+^ transients consistent with the biophysical properties of GCaMP were included in the analysis.

To identify cells that showed CS-evoked activity, we calculated intraday plasticity during the FC session by checking whether the average activity during CS presentation of trials 4 to 5 was higher than 2 SD above the average activity during trials 1 to 3. For the retrieval session, the same criterion was applied; here, the comparison was between the baseline period and the average of trials 1 to 5. For visualization purposes, calcium traces in fig. S5 were baselined to the mean of the 10 s preceding the CS presentation.

### Histology

After completion of the behavioral or tracing experiments, mice were transcardially perfused with 4% paraformaldehyde (PFA) in phosphate-buffered saline (PBS). For experiments involving rabies, perfusion was performed 5 to 8 days after injections. Brains were postfixed in PFA overnight at 4°C, cut with a VT1000S vibratome (Leica) with thickness of 80 to 120 μm, and imaged with AxioScan, Axioimager Z1, or LSM700 microscopes (Zeiss). The fiber tip positions, virus injection sites, and GRIN lens implantation sites were mapped against the mouse brain atlas (*43*). To identify starter neurons, sections were incubated with primary rabbit anti-2A peptide antibody (1:500; Merck Millipore, ABS31, lot no. 2746420) in 0.5% Triton X-100 in PBS for 48 hours at 4°C. Samples were rinsed three times with 0.1% Triton X-100 in PBS and then incubated overnight at 4°C with donkey anti-rabbit Alexa Fluor 568 (1:750; Thermo Fisher Scientific, A10042, lot no. 1757124) or donkey anti-rabbit Alexa Fluor 647 (1:750; Thermo Fisher Scientific, A31573, lot no. 1786284). Last, sections were washed four times with PBS, mounted on glass slides, and coverslipped. With a few exceptions, slices were stained with 4′,6-diamidino-2-phenylindole to facilitate area identification.

### Statistical analysis

Data are expressed as the means ± SEM unless stated otherwise. Box plots represent the median and the 25th and 75th percentiles, and their whiskers represent the data range. In some of the plots, outlier values are not shown for clarity of presentation, but all data points and animals were always included in the statistical analysis. A two-sided Wilcoxon rank sum test was used to compare two independent groups. For paired comparison, we used paired *t* test. Dunnett’s test was performed when more than two groups were compared against a control group. All the statistical test results were confirmed by bootstrapping analysis (fig. S6), which is more robust against non-normal sample distribution. Throughout the study, *P* < 0.05 was considered statistically significant. No statistical methods were used to predetermine sample sizes, but our sample sizes are similar to those generally used in the field. Freezing scoring for optogenetic manipulation experiments was performed blindly to experimental conditions.

## Supplementary Materials

### This PDF file includes:

Supplementary Materials Figs. S1 to S6 References

## Notes

View/request a protocol for this paper from *Bio-protocol*.
