# Supplementary material for: Disynaptic specificity of serial information flow for conditioned fear
Source: Sci Adv. 2023 Jan 18;9(3):eabq1637. doi: 10.1126/sciadv.abq1637 (PMC10957099; doi:10.1126/sciadv.abq1637)
Supplement: Supplementary file 1 — Supplementary Materials Figs. S1 to S6 References [file sciadv.abq1637_sm.pdf]

Science Advances
AAAS

Supplementary Materials for

Disynaptic specificity of serial information flow for conditioned fear

Léma Massi *et al.*

Corresponding author: Andreas Lüthi, andreas.luthi@fmi.ch

Sci. Adv. **9**, eabq1637 (2023)
DOI: 10.1126/sciadv.abq1637

This PDF file includes:

Supplementary Materials
Figs. S1 to S6
References

SUPPLYMENTARY MATERIALS

Supplementary Notes

In the experiments shown in **Fig. 2** and **Fig. 3**, where *SST-Cre* mice were used for di-synaptic tracing, our current virus combination design does not fully guarantee that only vlPAG-projecting SST+ CeA neurons serve as starter neurons for trans-synaptic rabies infection of BLAp neurons. Since there is local connectivity among SST+ neurons in the CeA (*21*), rabies viruses infecting axon terminals of SST+ CeA neurons in the vlPAG could also infect connected SST+ CeA neurons that do not project to vlPAG. As those secondarily infected CeA neurons are also transduced with TVA and G, they could, in turn, act as unintentional starter neurons for multi-synaptic labeling of BLAp neurons. Nevertheless, we think this potential pseudo-mono-synaptic labeling does not confound our findings for the following reasons.

First, we find relatively sparse rabies-positive neurons in the CeA and do not see dense overlap between 2A+ neurons and rabies-positive neurons in the CeA, suggesting that starter neurons are largely restricted to primarily infected vlPAG projecting SST+ neurons. This is consistent with the previous finding that only a fraction (approx. 30%) of SST+ CeA neurons project to vlPAG (*8*). Second, because synaptic connections between SST+ CeA neurons are inhibitory, a substantial contribution of tri- (or more) synaptic labeling would not be consistent with the effect of optogenetic manipulations of the SST+ di-synaptic pathway on behavior. Instead, we found that optogenetic manipulations of the SST+ disynaptic pathway had the same behavioral effect (**Fig. 2)** as non-cell-type-specific di-synaptic manipulations (**Fig. 1**), where starter neurons cannot be pseudo, thus excluding the above-mentioned scenario. Lastly, if there were frequent local trans-synaptic infections among SST+ CeA neurons, virtually the same populations in the CeA and BLA would be infected regardless of rabies injection site – CeA-targeted or vlPAG-targeted. However, we see different spatial distributions between the two cohorts in labeled BLAp neurons. Finally, photometry experiments showed clear differences in activity patterns between the mono-synaptically labeled and the di-synaptically labeled BLAp populations (**Fig. 3**). Thus, even though we cannot completely exclude the contribution of tri- (or more) synaptic labeling, our experimental strategy is sufficient to characterize the specificity of the BLAp-CeA(SST+)-vlPAG di-synaptic pathway.

In addition, in this study, we used TVA950, a very sensitive receptor for EnvA-pseudotyped RV, which is known to lead to local leak due to non-Cre-dependent low-level expression. Thus, the 2A-positive / RV-positive populations are not only starter cells – 2A-negative CeA neurons could work as starter cells. Thus, future studies aimed at histological quantification should rather use

TVA66, which is a less sensitive receptor suited for the quantification of rabies virus-based connectivity assay especially for locally dense circuits (*44*).

Supplementary Figures

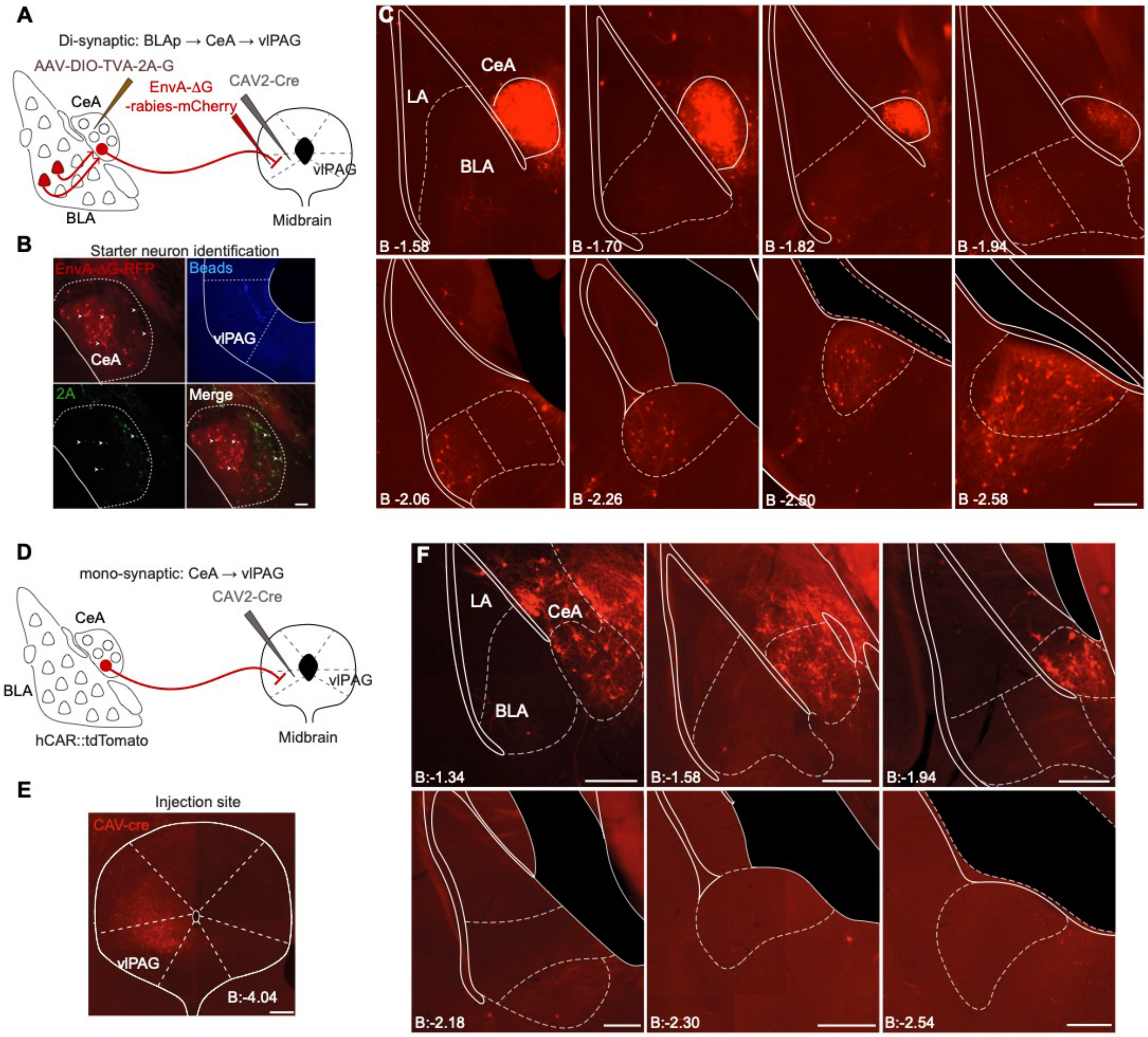

Fig. S1 | Di-synaptic connectivity from BLA to vlPAG.

A. Scheme illustrating viral injection strategy to express RFP in neurons projecting from BLA to the CeA-vlPAG-pathway. CAV2-Cre was injected into vlPAG and AAV-DIO-TVA-2A-G into CeA in hCAR mice. Then, rabies-RFP was injected into vlPAG.

B. Injection sites, CeA and vlPAG. vlPAG injections were marked by co-injecting blue beads. Starter neurons in the CeA were identified with immunohistochemistry for 2A. Scale bar: 50µm

C. Serial visualization of rabies-labeled neurons in the amygdala. Scale bar: 250µm

D. Scheme illustrating viral injection strategy to label neurons directly projecting to vlPAG. CAV2-Cre was injected into vlPAG in hCAR × tdTomato-reporter mice.

E. Injection site, vlPAG. Scale bar: 50µm

F. Serial visualization of labeled neurons in the amygdala. Unlike CeA neurons, BLA neurons do not directly project to vlPAG.

Scale bar: 250µm

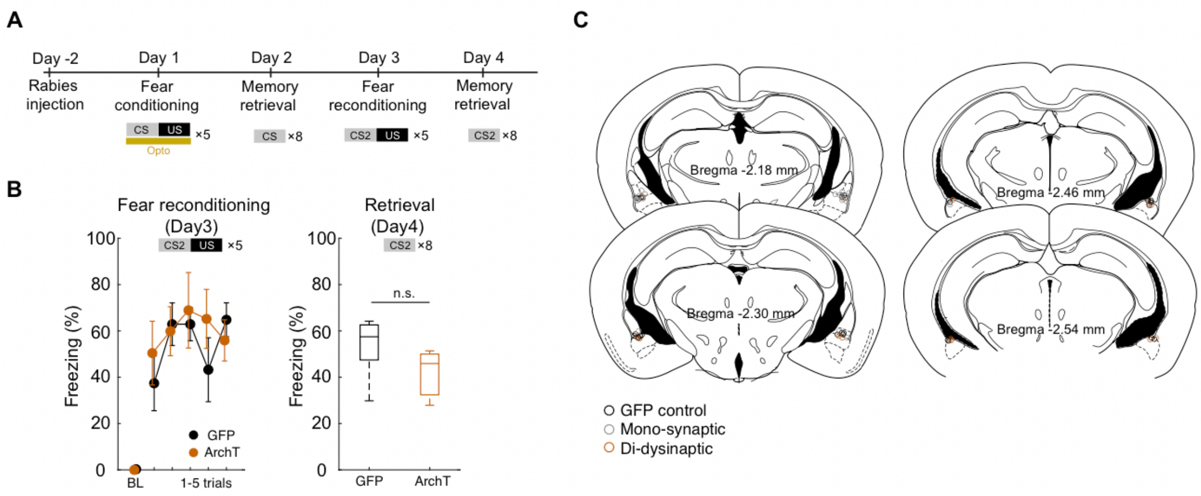

Fig. S2 | Reconditioning.

A. Experimental schedule of reconditioning experiments. After the initial FC and memory retrieval on Day1 and Day2, mice were again fear-conditioned using a novel CS – CS2.

B. Left: Average freezing levels (mean ± SEM) during reconditioning (Day3). Freezing levels during a 2 min baseline (BL) during each CS presentation (1-5 trials). N = 7,3 for GFP and ArchT, respectively. n.s.: P = 0.18; rank-sum test. Right: Average freezing levels (mean ± SEM) during 8 CS2 presentations during fear memory retrieval (Day4).

C. Optical fiber placements for experiments shown in **Fig. 1**.

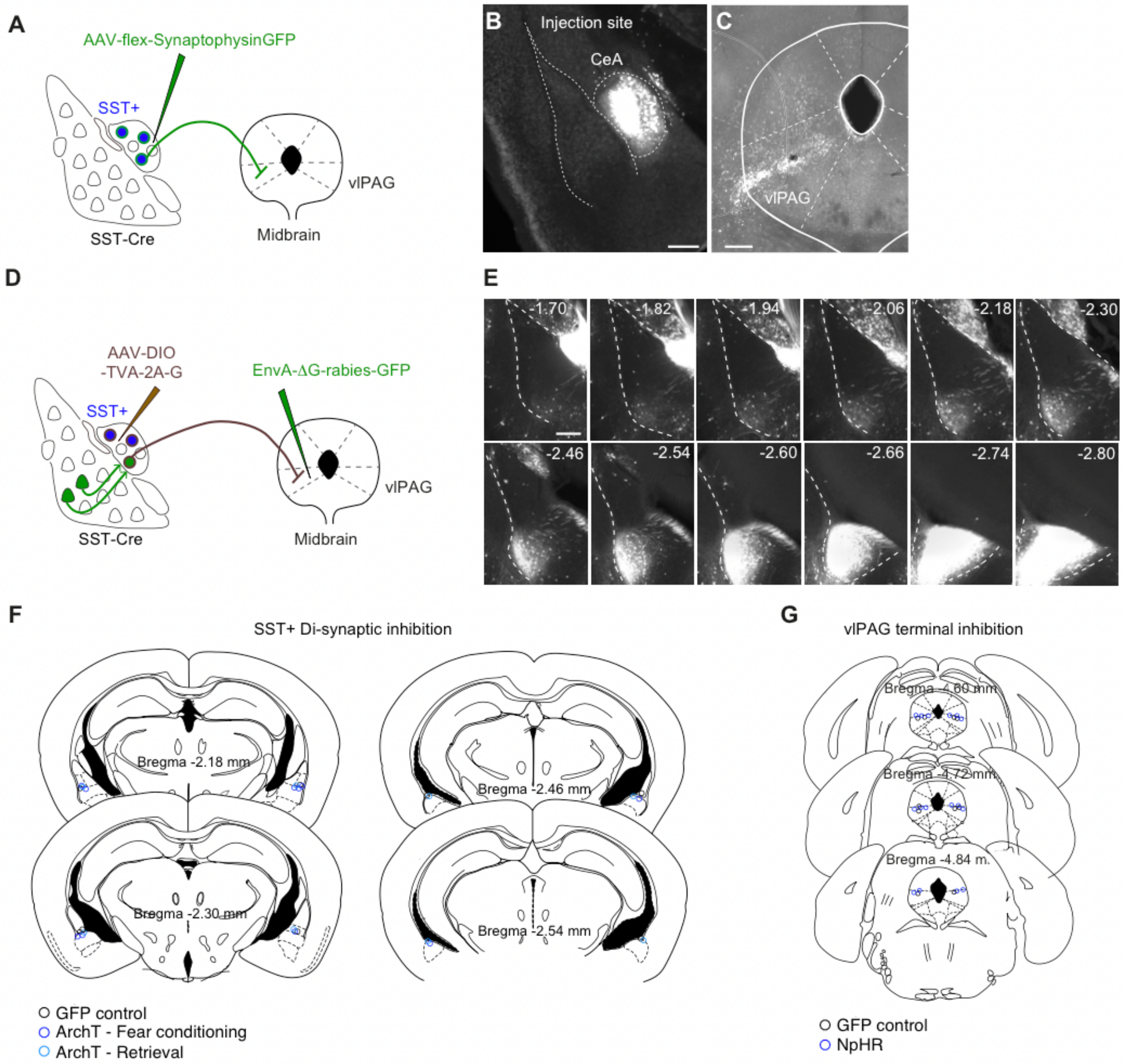

Fig. S3 | SST+ CeA neurons connect BLA to vlPAG.

A. Scheme illustrating viral injection strategy to express Synaptophysin-GFP in SST+ CeA neurons for axon terminal visualization.

B. Injection site, CeA.

C. SST+ CeA neurons project their axons to vlPAG. Scale bar: 250µm

D. Scheme illustrating viral injection strategy to express GFP in neurons projecting from BLA to CeA(SST+)-vlPAG-pathway. AAV-DIO-TVA-2A-G was injected into CeA in *SST-Cre* mice. Then, rabies-GFP was injected into vlPAG.

E. Serial visualization of labeled neurons in the amygdala. Similar to **Fig. S1C**, labeled neurons were preferentially found in posterolateral BLA. Scale bar: 250µm

F,G. Optical fiber placements for experiments shown in **Fig. 2**.

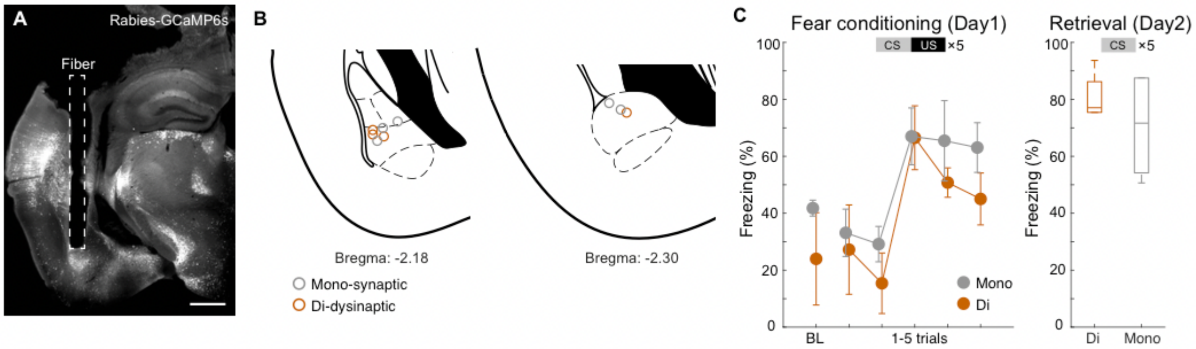

Fig. S4 | Additional data for fiber photometry recordings.

A. An example histological confirmation of the fiber implantation site.

B. Fiber placements.

C. Left: Average freezing levels (mean ± SEM) of implanted animals during conditioning (Day1). The 2 min baseline (BL) before the first CS presentation followed by the freezing levels during each CS presentation (1-5 trials). Right: Average freezing levels (mean ± SEM) during 5 CS presentations in fear memory retrieval (Day2). N = 4, 5 mice for Di (Di-synaptic rabies-GCaMP infected) and Mono (Mono-synaptic rabies-GCaMP infected) groups, respectively.

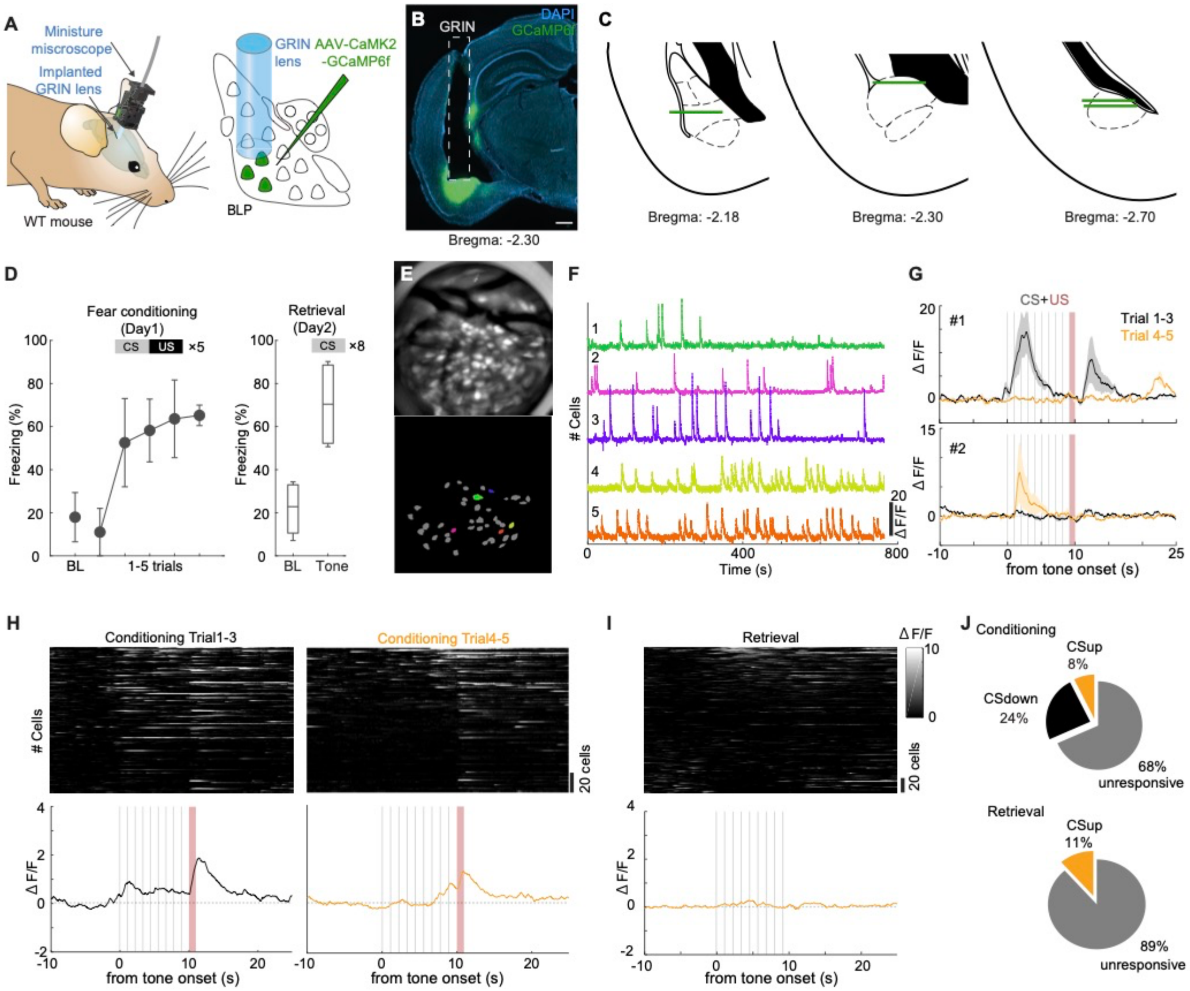

Fig. S5 | Cellular resolution calcium imaging revealed heterogeneous CS responses in posterolateral BLA neurons.

A. Scheme illustrating viral injection strategy to express GCaMP6f in neurons in posterolateral BLA. AAV-CaMK2-GCaMP6f was injected into BLA in BL6 WT mice, and then a GRIN lens was implanted targeting posterolateral BLA.

B. An example histological confirmation of the GRIN implantation site.

C. GRIN lens placements.

D. Left: Average freezing levels (mean ± SEM) of implanted animals during conditioning (Day1). The 2 min baseline (BL) before the first CS presentation, followed by the freezing levels during each CS presentation (1-5 trials). Right: Average freezing levels (mean ± SEM) during 8 CS presentations in fear memory retrieval (Day2). N = 4 mice.

E. Top: An example maximum intensity projection image of miniscope imaging. Bottom: Corresponding cell masks detected.

F. Five example calcium traces of neurons in **E** (color-matched) in the entire retrieval session (Day 2).

G. #1 and #2 in **F**, showing intrasession changes in CS responses – CSdown and Csup (see **MATERIALS AND METHODS**), respectively.

H, I. Responses of all recorded neurons show highly heterogeneous CS representations both during conditioning (**H**) and retrieval (**I**).

J. Fraction of CSup and CSdown neurons during conditioning (top) and retrieval (bottom) sessions.

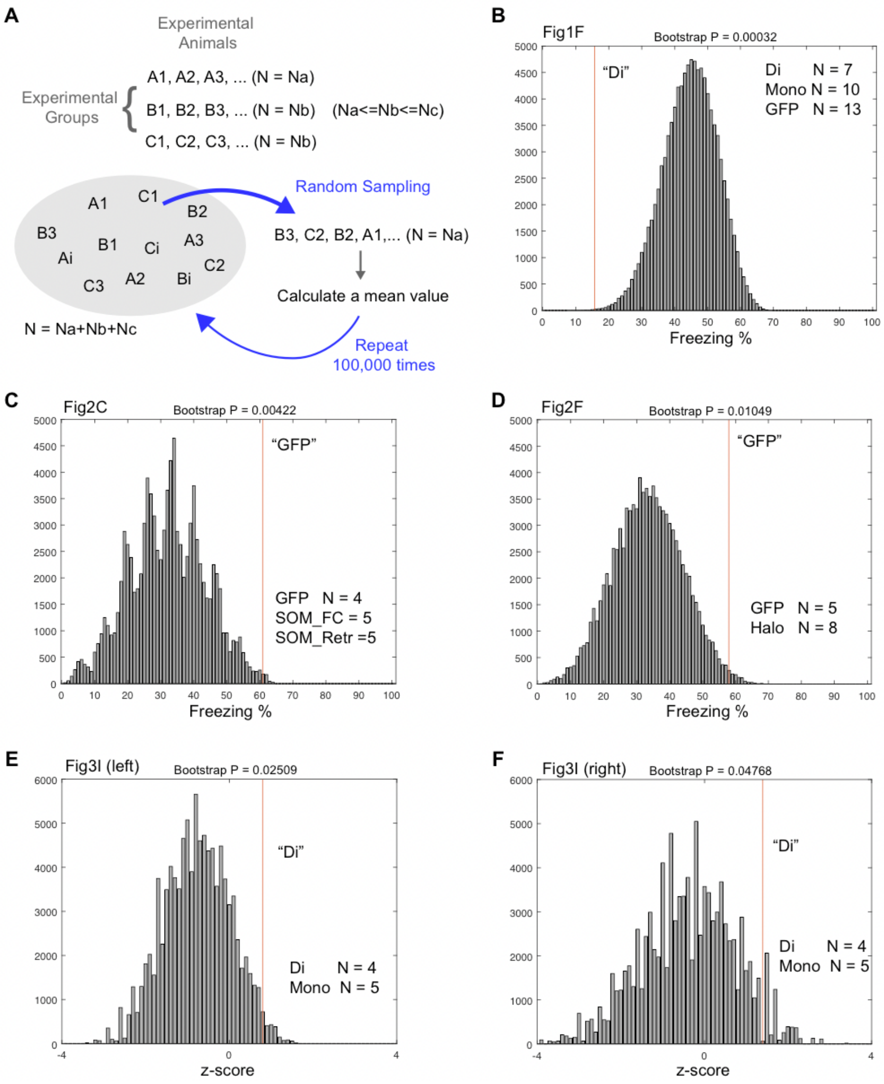

Fig. S6 | Bootstrapping for statistical analysis.
A. Scheme illustrating how across-group bootstrapping was performed. Samples from all the groups were mixed, samples of the smallest sample size were randomly sub-sampled, and then, a mean value was obtained. This procedure was repeated 100,000 times and distributions were estimated, with which bootstrap p values were obtained.
B. Analysis for **Fig. 1F.**
C. Analysis for **Fig. 2C.**
D. Analysis for **Fig. 2D.**
E. Analysis for **Fig. 3I** (left).
F. Analysis for **Fig. 3I** (right)**.**

REFERENCES

1. P. Tovote, J. P. Fadok, A. Lüthi, Neuronal circuits for fear and anxiety. *Nat. Rev. Neurosci.* **16**, 317–331 (2015).

2. A. Pitkänen, V. Savander, J. E. LeDoux, Organization of intra-amygdaloid circuitries in the rat: An emerging framework for understanding functions of the amygdala. *Trends Neurosci.* **20**, 517–523 (1997).

3. S. A. Jimenez, S. Maren, Nuclear disconnection within the amygdala reveals a direct pathway to fear. *Learn. Mem.* **16**, 766–768 (2009).

4. K. M. Tye, R. Prakash, S.-Y. Kim, L. E. Fenno, L. Grosenick, H. Zarabi, K. R. Thompson, V. Gradinaru, C. Ramakrishnan, K. Deisseroth, Amygdala circuitry mediating reversible and bidirectional control of anxiety. *Nature* **471**, 358–362 (2011).

5. P. Namburi, A. Beyeler, S. Yorozu, G. G. Calhoon, S. A. Halbert, R. Wichmann, S. S. Holden, K. L. Mertens, M. Anahtar, A. C. Felix-Ortiz, I. R. Wickersham, J. M. Gray, K. M. Tye, A circuit mechanism for differentiating positive and negative associations. *Nature* **520**, 675–678 (2015).

6. J. E. LeDoux, J. Iwata, P. Cicchetti, D. J. Reis, Different projections of the central amygdaloid nucleus mediate autonomic and behavioral correlates of conditioned fear. *J. Neurosci.* **8**, 2517–2529 (1988).

7. M. S. Fanselow, The midbrain periaqueductal gray as a coordinator of action in response to fear and anxiety, in *The Midbrain Periaqueductal Gray Matter: Functional, Anatomical, and Neurochemical Organization*, A. Depaulis, R. Bandler, Eds. (Springer US, 1991), pp. 151–173.

8. M. A. Penzo, V. Robert, B. Li, Fear conditioning potentiates synaptic transmission onto long-range projection neurons in the lateral subdivision of central amygdala. *J. Neurosci.* **34**, 2432–2437 (2014).

9. P. Tovote, M. S. Esposito, P. Botta, F. Chaudun, J. P. Fadok, M. Markovic, S. B. E. Wolff, C. Ramakrishnan, L. Fenno, K. Deisseroth, C. Herry, S. Arber, A. Lüthi, Midbrain circuits for defensive behaviour. *Nature* **534**, 206–212 (2016).

10. B. Li, Central amygdala cells for learning and expressing aversive emotional memories. *Curr. Opin. Behav. Sci.* **26**, 40–45 (2019).

11. R. D. Palmiter, The parabrachial nucleus: CGRP neurons function as a general alarm. *Trends Neurosci.* **41**, 280–293 (2018).

12. M. Nagase, K. Mikami, A. M. Watabe, Parabrachial-to-amygdala control of aversive learning. *Curr. Opin. Behav. Sci.* **26**, 18–24 (2019).

13. J. Kim, X. Zhang, S. Muralidhar, S. A. LeBlanc, S. Tonegawa, Basolateral to central amygdala neural circuits for appetitive behaviors. *Neuron* **93**, 1464–1479.e5 (2017).

14. J. Kim, M. Pignatelli, S. Xu, S. Itohara, S. Tonegawa, Antagonistic negative and positive neurons of the basolateral amygdala. *Nat. Neurosci.* **19**, 1636–1646 (2016).

15. H. Hintiryan, I. Bowman, D. L. Johnson, L. Korobkova, M. Zhu, N. Khanjani, L. Gou, L. Gao, S. Yamashita, M. S. Bienkowski, L. Garcia, N. N. Foster, N. L. Benavidez, M. Y. Song, D. Lo, K. R. Cotter, M. Becerra, S. Aquino, C. Cao, R. P. Cabeen, J. Stanis, M. Fayzullina, S. A. Ustrell, T. Boesen, A. J. Tugangui, Z.-G. Zhang, B. Peng, M. S. Fanselow, P. Golshani, J. D. Hahn, I. R. Wickersham, G. A. Ascoli, L. I. Zhang, H.-W. Dong, Connectivity characterization of the mouse basolateral amygdalar complex. *Nat. Commun.* **12**, 1–25 (2021).

16. J. E. Krettek, J. L. Price, A description of the amygdaloid complex in the rat and cat with observations on intra-amygdaloid axonal connections. *J. Comp. Neurol.* **178**, 255–279 (1978).

17. D. Paré, Y. Smith, J. F. Paré, Intra-amygdaloid projections of the basolateral and basomedial nuclei in the cat: Phaseolus vulgaris-leucoagglutinin anterograde tracing at the light and electron microscopic level. *Neuroscience* **69**, 567–583 (1995).

18. C. Xu, S. Krabbe, J. Gründemann, P. Botta, J. P. Fadok, F. Osakada, D. Saur, B. F. Grewe, M. J. Schnitzer, E. M. Callaway, A. Lüthi, Distinct hippocampal pathways mediate dissociable roles of context in memory retrieval. *Cell* **167**, 961–972.e16 (2016).

19. J. P. Fadok, S. Krabbe, M. Markovic, J. Courtin, C. Xu, L. Massi, P. Botta, K. Bylund, C. Müller, A. Kovacevic, P. Tovote, A. Lüthi, A competitive inhibitory circuit for selection of active and passive fear responses. *Nature* **542**, 96–100 (2017).

20. H. Li, M. A. Penzo, H. Taniguchi, C. D. Kopec, Z. J. Huang, B. Li, Experience-dependent modification of a central amygdala fear circuit. *Nat. Neurosci.* **16**, 332–339 (2013).

21. S. Hunt, Y. Sun, H. Kucukdereli, R. Klein, P. Sah, Intrinsic circuits in the lateral central amygdala. *eNeuro* **4**, ENEURO.0367-6.2017 (2017).

22. B. F. Grewe, J. Gründemann, L. J. Kitch, J. A. Lecoq, J. G. Parker, J. D. Marshall, M. C. Larkin, P. E. Jercog, F. Grenier, J. Z. Li, A. Lüthi, M. J. Schnitzer, Neural ensemble dynamics underlying a long-term associative memory. *Nature* **543**, 670–675 (2017).

23. K. M. Hagihara, O. Bukalo, M. Zeller, A. Aksoy-Aksel, N. Karalis, A. Limoges, T. Rigg, T. Campbell, A. Mendez, C. Weinholtz, M. Mahn, L. S. Zweifel, R. D. Palmiter, I. Ehrlich, A. Lüthi, A. Holmes, Intercalated amygdala clusters orchestrate a switch in fear state. *Nature* **594**, 403–407 (2021).

24. T. R. Reardon, A. J. Murray, G. F. Turi, C. Wirblich, K. R. Croce, M. J. Schnell, T. M. Jessell, A. Losonczy, Rabies virus CVS-N2c(ΔG) strain enhances retrograde synaptic transfer and neuronal viability. *Neuron* **89**, 711–724 (2016).

25. S. Chatterjee, H. A. Sullivan, B. J. MacLennan, R. Xu, Y. Hou, T. K. Lavin, N. E. Lea, J. E. Michalski, K. R. Babcock, S. Dietrich, G. A. Matthews, A. Beyeler, G. G. Calhoon, G. Glober, J. D. Whitesell, S. Yao, A. Cetin, J. A. Harris, H. Zeng, K. M. Tye, R. C. Reid, I. R. Wickersham, Nontoxic, double-deletion-mutant rabies viral vectors for retrograde targeting of projection neurons. *Nat. Neurosci.* **21**, 638–646 (2018).

26. E. Ciabatti, A. González-Rueda, L. Mariotti, F. Morgese, M. Tripodi, Life-long genetic and functional access to neural circuits using self-inactivating rabies virus. *Cell* **170**, 382–392.e14 (2017).

27. W. Haubensak, P. S. Kunwar, H. Cai, S. Ciocchi, N. R. Wall, R. Ponnusamy, J. Biag, H.-W. Dong, K. Deisseroth, E. M. Callaway, M. S. Fanselow, A. Lüthi, D. J. Anderson, Genetic dissection of an amygdala microcircuit that gates conditioned fear. *Nature* **468**, 270–276 (2010).

28. Y. Wang, S. Krabbe, M. Eddison, F. E. Henry, G. Fleishman, A. L. Lemire, L. Wang, W. Korff, P. W. Tillberg, A. Lüthi, S. M. Sternson, Multimodal mapping of cell types and projections in the central nucleus of the amygdala. bioRxiv 2022.10.19.512845 [**Preprint**]. 22 October 2022. https://doi.org/10.1101/2022.10.19.512845.

29. L. A. Schwarz, K. Miyamichi, X. J. Gao, K. T. Beier, B. Weissbourd, K. E. DeLoach, J. Ren, S. Ibanes, R. C. Malenka, E. J. Kremer, L. Luo, Viral-genetic tracing of the input-output organization of a central noradrenaline circuit. *Nature* **524**, 88–92 (2015).

30. H. Taniguchi, M. He, P. Wu, S. Kim, R. Paik, K. Sugino, D. Kvitsiani, Y. Fu, J. Lu, Y. Lin, G. Miyoshi, Y. Shima, G. Fishell, S. B. Nelson, Z. J. Huang, A resource of Cre driver lines for genetic targeting of GABAergic neurons in cerebral cortex. *Neuron* **71**, 995–1013 (2011).

31. T. Tallone, S. Malin, A. Samuelsson, J. Wilbertz, M. Miyahara, K. Okamoto, L. Poellinger, L. Philipson, S. Pettersson, A mouse model for adenovirus gene delivery. *Proc. Natl. Acad. Sci. U.S.A.* **98**, 7910–7915 (2001).

32. F. Osakada, E. M. Callaway, Design and generation of recombinant rabies virus vectors. *Nat. Protoc.* **8**, 1583–1601 (2013).

33. A. Wertz, S. Trenholm, K. Yonehara, D. Hillier, Z. Raics, M. Leinweber, G. Szalay, A. Ghanem, G. Keller, B. Rózsa, K.-K. Conzelmann, B. Roska, PRESYNAPTIC NETWORKS. Single-cell-initiated monosynaptic tracing reveals layer-specific cortical network modules. *Science* **349**, 70–74 (2015).

34. F. Osakada, T. Mori, A. H. Cetin, J. H. Marshel, B. Virgen, E. M. Callaway, New rabies virus variants for monitoring and manipulating activity and gene expression in defined neural circuits. *Neuron* **71**, 617–631 (2011).

35. I. R. Wickersham, D. C. Lyon, R. J. O. Barnard, T. Mori, S. Finke, K.-K. Conzelmann, J. A. T. Young, E. M. Callaway, Monosynaptic restriction of transsynaptic tracing from single, genetically targeted neurons. *Neuron* **53**, 639–647 (2007).

36. C. Soudais, C. Laplace-Builhe, K. Kissa, E. J. Kremer, Preferential transduction of neurons by canine adenovirus vectors and their efficient retrograde transport in vivo. *FASEB J.* **15**, 2283–2285 (2001).

37. S. Krabbe, E. Paradiso, S. d'Aquin, Y. Bitterman, J. Courtin, C. Xu, K. Yonehara, M. Markovic, C. Müller, T. Eichlisberger, J. Gründemann, F. Ferraguti, A. Lüthi, Adaptive disinhibitory gating by VIP interneurons permits associative learning. *Nat. Neurosci.* **22**, 1834–1843 (2019).

38. E. Pecho-Vrieseling, M. Sigrist, Y. Yoshida, T. M. Jessell, Specificity of sensory–motor connections encoded by Sema3e–Plxnd1 recognition. *Nature* **459**, 842–846 (2009).

39. T.-W. Chen, T. J. Wardill, Y. Sun, S. R. Pulver, S. L. Renninger, A. Baohan, E. R. Schreiter, R. A. Kerr, M. B. Orger, V. Jayaraman, L. L. Looger, K. Svoboda, D. S. Kim, Ultrasensitive fluorescent proteins for imaging neuronal activity. *Nature* **499**, 295–300 (2013).

40. G. Lopes, N. Bonacchi, J. Frazão, J. P. Neto, B. V. Atallah, S. Soares, L. Moreira, S. Matias, P. M. Itskov, P. A. Correia, R. E. Medina, L. Calcaterra, E. Dreosti, J. J. Paton, A. R. Kampff, Bonsai: An event-based framework for processing and controlling data streams. *Front. Neuroinform.* **9**, 7 (2015).

41. M. Guizar-Sicairos, S. T. Thurman, J. R. Fienup, Efficient subpixel image registration algorithms. *Opt. Lett. OL.* **33**, 156–158 (2008).

42. P. Zhou, S. L. Resendez, J. Rodriguez-Romaguera, J. C. Jimenez, S. Q. Neufeld, A. Giovannucci, J. Friedrich, E. A. Pnevmatikakis, G. D. Stuber, R. Hen, M. A. Kheirbek, B. L.

Sabatini, R. E. Kass, L. Paninski, Efficient and accurate extraction of in vivo calcium signals from microendoscopic video data. *eLife* **7**, e28728 (2018).

43. G. Paxinos, K. B. J. Franklin, *The Mouse Brain in Stereotaxic Coordinates* (Academic, 2001).

44. K. Miyamichi, Y. Shlomai-Fuchs, M. Shu, B. C. Weissbourd, L. Luo, A. Mizrahi, Dissecting local circuits: Parvalbumin interneurons underlie broad feedback control of olfactory bulb output. *Neuron* **80**, 1232–1245 (2013).
